# Bactericidal effects and accelerated wound healing using Tb_4_O_7_ nanoparticles with intrinsic oxidase-like activity

**DOI:** 10.1186/s12951-019-0487-x

**Published:** 2019-04-16

**Authors:** Chen Li, Yurong Sun, Xiaoping Li, Sanhong Fan, Yimin Liu, Xiumei Jiang, Mary D. Boudreau, Yue Pan, Xin Tian, Jun-Jie Yin

**Affiliations:** 1 School for Life Science, Shanxi University, Taiyuan, 030006 China; 2 Guangdong Provincial Key Laboratory of Malignant Tumor Epigenetics and Gene Regulation, Department of Radiation Oncology, Medical Research Center, Sun Yat-Sen Memorial Hospital, Sun Yat-Sen University, Guangzhou, 510120 China; 3 Division of Analytical Chemistry, Office of Regulatory Science, Center for Food Safety and Applied Nutrition, U.S. Food and Drug Administration, College Park, MD 20740 USA; 4 Division of Biochemical Toxicology, National Center for Toxicological Research, U.S. Food and Drug Administration, Jefferson, AR 72079 USA; 5 State Key Laboratory of Radiation Medicine and Protection, School for Radiological and Interdisciplinary Sciences (RAD-X), Collaborative Innovation Center of Radiation Medicine of Jiangsu Higher Education Institutions, Soochow University, Suzhou, 215123 China

**Keywords:** Tb_4_O_7_ nanoparticles, Oxidase, Reactive oxygen species, Antibacterial, Wound healing

## Abstract

**Background:**

Nanomaterials that exhibit intrinsic enzyme-like characteristics have shown great promise as potential antibacterial agents. However, many of them exhibit inefficient antibacterial activity and biosafety problems that limit their usefulness. The development of new nanomaterials with good biocompatibility and rapid bactericidal effects is therefore highly desirable. Here, we show a new type of terbium oxide nanoparticles (Tb_4_O_7_ NPs) with intrinsic oxidase-like activity for in vitro and in vivo antibacterial application.

**Results:**

We find that Tb_4_O_7_ NPs can quickly oxidize a series of organic substrates in the absence of hydrogen peroxide. The oxidase-like capacity of Tb_4_O_7_ NPs allows these NPs to consume antioxidant biomolecules and generate reactive oxygen species to disable bacteria in vitro. Moreover, the in vivo experiments showed that Tb_4_O_7_ NPs are efficacious in wound-healing and are protective of normal tissues.

**Conclusions:**

Our results reveal that Tb_4_O_7_ NPs have intrinsic oxidase-like activity and show effective antibacterial ability both in vitro and in vivo. These findings demonstrate that Tb_4_O_7_ NPs are effective antibacterial agents and may have a potential application in wound healing.

**Electronic supplementary material:**

The online version of this article (10.1186/s12951-019-0487-x) contains supplementary material, which is available to authorized users.

## Background

Wound infection is an important cause of poor wound healing and its treatment often requires the use of antibiotics [1, 2]. However, excessive use of antibiotics may lead to the development of antibiotic-resistant bacteria, and may also cause side effects on human health, such as gastrointestinal disturbances. In recent years, developments in nanomaterial technology have provided an opportunity to develop novel antimicrobial agents. Due to the diversity in mechanisms of action against bacteria, bacterial cells are less likely to develop antibacterial resistance compared to existing antibiotics [3–5]. However, most of these nanomaterials have application limitations, such as cytotoxicity, not biocompatible for human use, and environmental concerns.

Nanozymes are nanomaterials that catalyze the same reactions originally catalyzed by natural enzymes in biological systems [6–8]. Over the past several years, a wide variety of nanomaterials, such as noble metals [9–11], metal oxides [12–14], and carbon nanomaterials [15–18], have been explored as potential nanozymes. Based on their intrinsic enzyme-like activity, several nanozymes have been used in antibacterial applications [19–23]. For instance, platinum nanomaterials have shown effective antibacterial activity in the presence of hydrogen peroxide (H_2_O_2_) [24]. The antibacterial activity of these nanozymes are attributed primarily to their oxidase- and peroxidase-like activities that catalyze the production of hydroxyl radicals (·OH) in the presence of exogenous H_2_O_2_ and enhance the cellular levels of reactive oxygen species (ROS) within bacteria cells. Fang et al. also showed that palladium nanomaterials with oxidase- and peroxidase-like activities displayed effective antibacterial activity in the presence of H_2_O_2_ [25]. Although many reported enzyme-like nanomaterials have been proposed as novel antibacterial agents, the high price and persistence in living tissues are still important issues. Moreover, the application of H_2_O_2_ in human wound disinfection is harmful to healthy tissue and may delay wound healing [26].

Terbium oxide nanoparticles (Tb_4_O_7_ NPs) have been extensively used as precursors for the synthesis of lanthanide nanophosphors and superconductor materials [27, 28]. For example, Tb_4_O_7_ complexed with reduced graphene oxide composite exhibit typical green emission of terbium ions as well as the blue self-luminescence of graphene [28]. In addition, it has been found that Tb_4_O_7_ NPs can be used as analytical reagents for food analysis [29]. Compared with noble metal nanomaterials, Tb_4_O_7_ NPs are easier to synthesize and are less expensive. However, a review of scientific literature was unable to find any studies that described the enzyme-like activity of Tb_4_O_7_ NPs and their applications as antibacterial agents. In this paper, we show that Tb_4_O_7_ NPs have an intrinsic oxidase-like activity at acidic pH values, as they quickly oxidize a series of organic substrates in the absence of H_2_O_2_. We then demonstrate the relationship between the oxidase-like property of Tb_4_O_7_ NPs and their antibacterial activity with in vitro studies. Finally, the effects of Tb_4_O_7_ NPs on wound disinfection and healing are evaluated in in vivo studies using a wound infection mouse model.

## Materials and methods

### Chemicals and materials

Tb_4_O_7_ NPs were purchased from US Research Nanomaterials, Inc. (TX, USA). 3,3,5,5-tetramethylbenzidinedihydrochloride (TMB), diammonium 2,2′-azino-bis(3-ethylbenzothiazoline-6-sulfonate) (ABTS), *o*-phenylenediamine (OPD), and Lipid Peroxidation MDA Assay Kit were all purchased from Sigma-Aldrich (St. Louis, MO). The Live/Dead BacLight bacterial viability kit and 2′,7′-dichlorodihydrofluorescein diacetate (DCFH-DA) were obtained from Thermo Fisher Scientific, Inc. (MA, USA). 5-tert-butoxycarbonyl-5-methyl-1-pyrroline-*N*-oxide (BMPO) and Cell Counting Kit-8 (CCK-8) were obtained from Dojindo Laboratories (Kumamoto, Japan). *Escherichia coli* (*E. coli*) and *Staphylococcus aureus* subsp. *aureus* (*S. aureus*) were obtained from the China General Microbiological Culture Collection Center (CGMCC, Beijing, China). Human umbilical vein endothelial cells (HUVECs) were purchased from the American Type Culture Collection (ATCC, MD, USA).

### Characterization of Tb_4_O_7_ NPs

The hydrodynamic size and zeta potential of the Tb_4_O_7_ NPs were measured using a Zetasizer Nano-ZS (Malvern, UK). The morphology and size of the Tb_4_O_7_ NPs were characterized using a transmission electron microscopy (TEM, Tecnai G-20, FEI). The UV–vis absorption spectrum was recorded on a spectrophotometer (UV-3600, Shimadzu).

### Electron spin resonance spectroscopic measurements

The electron spin resonance (ESR) measurements were carried out using a Bruker EMX ESR spectrometer according to our previous study [3, 9]. The final concentration of each component is described in each figure caption. All the ESR measurements were carried out at ambient temperature.

### Measurement of intracellular ROS

After Tb_4_O_7_ NPs (100 μg/mL) treatment, bacteria (1 × 10^9^ CFU/mL) were collected by centrifugation and incubated with DCFH-DA (10 µM) for 30 min at dark, and stained bacteria were visualized with a confocal laser microscopy.

### Cytotoxicity experiments

HUVECs were employed for investigating the cytotoxicity of Tb_4_O_7_ NPs. HUVECs were seeded at a density of 1 × 10^5^ cells/well in 96-well plates and incubated overnight. The HUVECs were then incubated with Tb_4_O_7_ NPs (0–100 μg/mL) for 24 h, and cell viability was measured by MTT assay.

### Mice injury model

BALB/c mice (8 weeks) were purchased from Pengsheng. On the day 0, the mice were anesthetized using 10% chloral hydrate. Then, the dorsal hair of mouse was shaved, full-thickness skin wounds with the diameter of 10 mm were created on the back of each mouse. After 24 h (day 1), the mice were treated with 50 μL PBS or Tb_4_O_7_ NPs (100 μg/mL). The Tb_4_O_7_ NPs were dripping on the surface of the wound.

### Hemolysis test

Fresh blood was collected under sterile conditions from healthy BALB/c mice (n = 5) into an anticoagulation tube. The red blood cells were precipitated by centrifugation at 2000 rpm for 10 min and washed three times with PBS buffer solution to obtain red blood cells. The appropriate amount of red blood cells was diluted five times with PBS buffer solution to prepare a red blood cell solution. 20 μL of the diluted red blood cell suspension was mixed with a series of different concentrations of Tb_4_O_7_ NPs (0–200 μg/mL); ultrapure water was used as control. All the above samples were incubated at 37 °C for 2 h, centrifuged at 2000 rpm for 10 min, imaged, and the supernatant after centrifugation was taken in a 96-well plate to measure the absorbance at 540 nm using a microplate reader. The hemolysis rate was calculated as follows:

$${\text{Hemolysis rate }}\left( \% \right) = \left( {{\text{sample absorption}} - {\text{negative control absorption}}} \right)/\left( {{\text{positive control absorption}} - {\text{negative control absorption}}} \right) \times 100\% ,{\text{ and hemolysis rate exceeding 5}}\% {\text{is considered hemolysis}}.$$

## Results and discussion

### Characterization of Tb_4_O_7_ NPs

The Tb_4_O_7_ NPs used in the present study were purchased from US Research Nanomaterials, Inc. The physical characterization of Tb_4_O_7_ NPs is shown in Additional file 1: Figure S1 and included images of particle core size and shape captured by TEM, the mean and homogeneity of particle hydrodynamic size by dynamic light scattering (DLS), and particle absorption spectrum by UV–vis. According to TEM and DLS data, the dispersity of Tb_4_O_7_ NPs is poor. The mean core particle size of Tb_4_O_7_ NPs is approximately 200 nm (Additional file 1: Figure S1a, b); while the DLS result of Tb_4_O_7_ NPs is around 400 nm (Additional file 1: Figure S1c). This is mainly due to the size measured by DLS was a hydrodynamic size, and therefore the nanoparticles showed a larger hydrodynamic volume due to solvent effect in the hydrated state. The zeta potential value of Tb_4_O_7_ NPs is 31.6 mV in water. The UV–vis spectrum of Tb_4_O_7_ NPs is shown in Additional file 1: Figure S1d.

### Catalytic activity of Tb_4_O_7_ NPs as oxidase mimetics

**Fig. 1:**
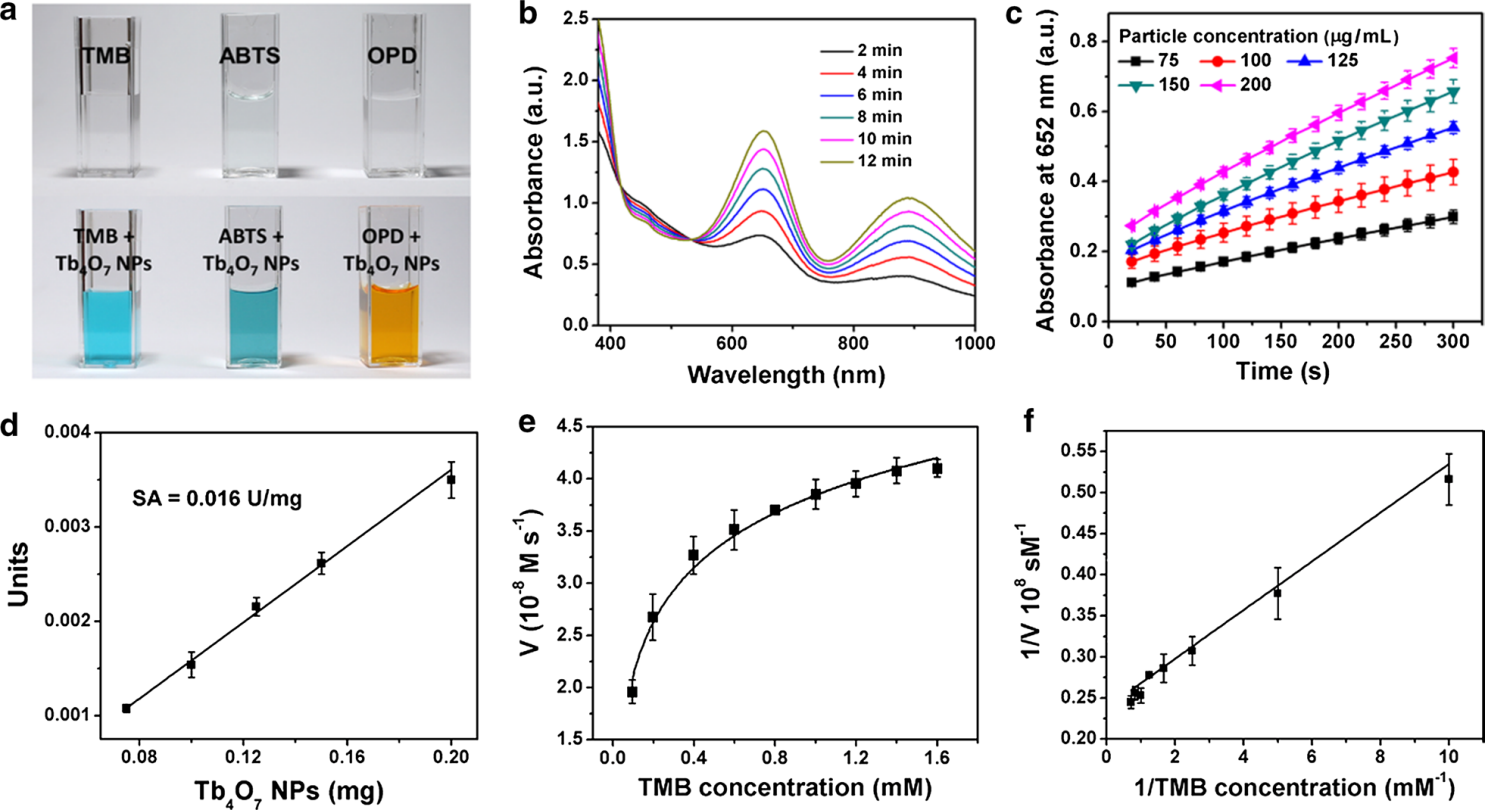
Oxidase-like activity of Tb_4_O_7_ NPs. **a** A photograph showing the capability of the Tb_4_O_7_ NPs in catalyzing the oxidations of TMB, ABTS, and OPD that produce colored products. **b** Time-dependent absorption spectra of TMB catalyzed by Tb_4_O_7_ NPs. **c** Absorbance at 652 nm measured from samples containing TMB and different concentrations of Tb_4_O_7_ NPs. **d** The specific activities of Tb_4_O_7_ NPs. Steady-state kinetic assays of Tb_4_O_7_ NPs (**e**, **f**). **e** TMB concentration dependence of initial reaction velocity. **f** Double-reciprocal plot generated from (**d**)

The oxidase-like activity of Tb_4_O_7_ NPs was evaluated using the substrate TMB. The UV–vis spectroscopy measurements show time-dependent increases in TMB oxidation catalyzed by Tb_4_O_7_ NPs, yielding a blue-colored product (Fig. 1a, b). In addition, Tb_4_O_7_ NPs can also catalyze the oxidation of ABTS and OPD (Fig. 1a).

We used the oxidation of TMB as a model reaction and found that the catalytic efficiency of the Tb_4_O_7_ NPs is dependent on TMB concentrations, pH and temperature (Fig. 1c and Additional file 1: Figure S2). As shown in Additional file 1: Figure S2a, Tb_4_O_7_ NPs exhibit excellent catalytic activity over a broad temperature range (25–60 °C). Moreover, an acidic condition (pH = 3.6) is conducive to the oxidase-like activity of Tb_4_O_7_ NPs (Additional file 1: Figure S2b). We adopted pH 3.6 and 25 °C (room temperature) as the standard conditions for subsequent studies.

Next, we determined the apparent steady-state kinetic parameters for the reaction of Tb_4_O_7_ NPs with TMB. Typical Michaelis–Menten curves were established (Fig. 1d). The curves were then fitted to the double-reciprocal Lineweaver–Burk plots (Fig. 1e), from which the kinetic parameters shown in Table 1 were determined.

**Table 1:** The kinetic constants of Tb_4_O_7_ NPs

	[E] (M)	*K*_m_ (M)	*V*_max_ (M/s)	*K*_cat_ (s^−1^)	*K*_cat_/*K*_m_ (M^−1^ S^−1^)
Tb_4_O_7_ NPs	7.04 × 10^−10^	1.24 × 10^−4^	4.31 × 10^−8^	1.61 × 10^−4^	1.30

[E] is the concentration of Tb_4_O_7_ NPs. The particle number of Tb_4_O_7_ NPs is calculated using the density and diameter of Tb_4_O_7_ NPs. Dividing the particle number by the Avogadro constant is the molar concentration of Tb_4_O_7_ NPs

### Effects of Tb_4_O_7_ NPs on the anti-oxidant defense system

**Fig. 2:**
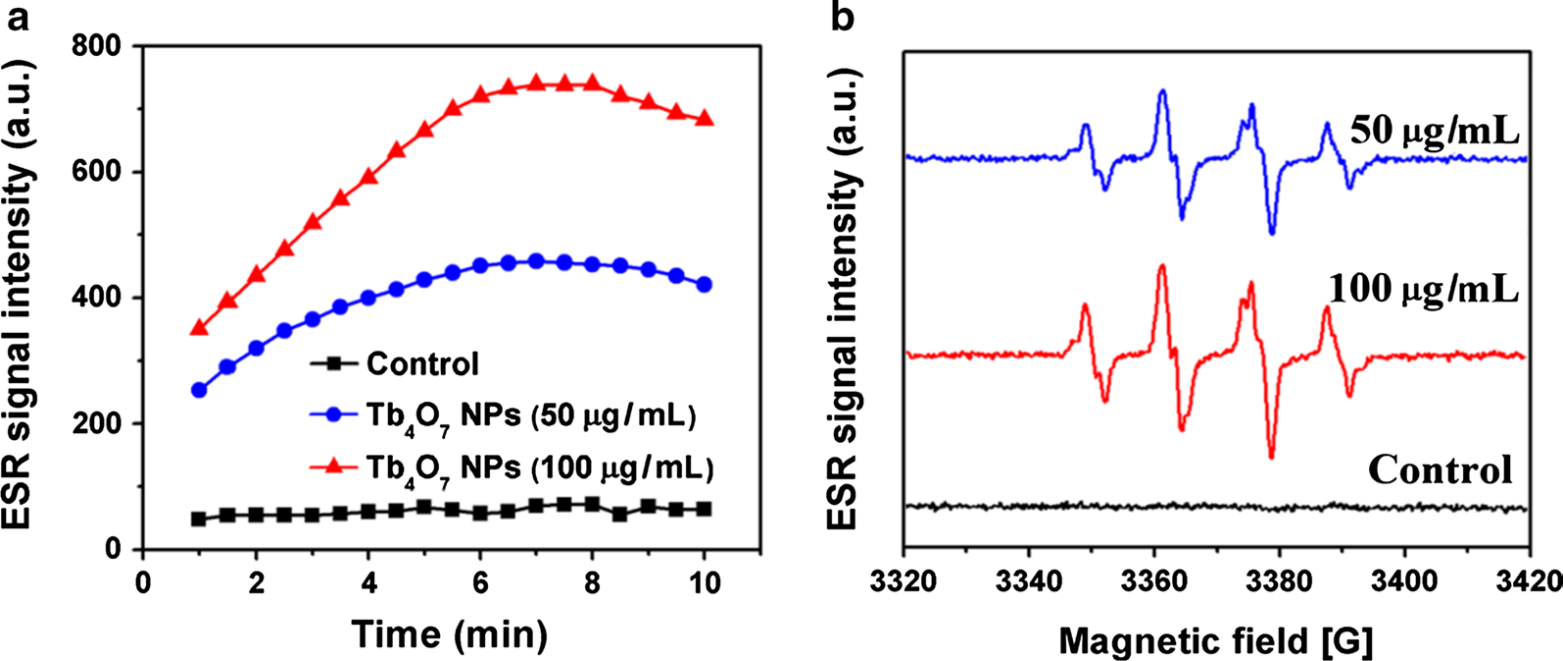
**a** Oxidation of AA by Tb_4_O_7_ NPs. **b** ESR spectra of BMPO/·OH generated from a sample solution containing 25 mM BMPO, 1 mM H_2_O_2_ in the absence (control) and presence of different concentrations of Tb_4_O_7_ NPs

The above results show that Tb_4_O_7_ NPs have oxidase-like activity oxidizing TMB, ABTS, and OPD in the absence of H_2_O_2_. We predict that Tb_4_O_7_ NPs would deplete intracellular antioxidants and, eventually, disrupt the antioxidant defense systems of bacteria. To test this hypothesis, we examined the effects of Tb_4_O_7_ NPs on ascorbic acid (AA) oxidation in vitro. AA is an important endogenous bacterial antioxidant that prevents cellular damage from ROS. AA can be oxidized to form an intermediate ascorbyl radical (·AA), which is detectable by ESR spectroscopy [9]. As shown in Fig. 2a, the oxidation of AA was negligible within 10 min, while in the presence of Tb_4_O_7_ NPs the system showed a time-dependent increase in the ESR signal intensity in the first 8 min and then decreased over time. These results indicate that Tb_4_O_7_ NPs can accelerate AA oxidation.

In the substrate oxidation mechanism of most oxidases in nature, oxygen acts as the electron acceptor and is reduced to H_2_O_2_. To gain a better understanding of the oxidation of AA by Tb_4_O_7_ NPs, we examined whether the catalytic oxidation product of Tb_4_O_7_ NPs and AA produced H_2_O_2_. As shown in Additional file 1: Figure S3, a marked increase of H_2_O_2_ was detected in the presence of Tb_4_O_7_ compared to control (AA alone). Moreover, the production of H_2_O_2_ was Tb_4_O_7_ concentration-dependent.

Previous studies have demonstrated that several kinds of nanoparticles are capable of catalyzing the production of hydroxyl radicals by H_2_O_2_ [30, 31]. Therefore, we determined whether Tb_4_O_7_ NPs would catalyze the production of hydroxyl radicals by H_2_O_2_ using ESR spectroscopy. We selected BMPO as the capture agent, since BMPO can capture hydroxyl radicals to form BMPO/·OH adducts indicated by the presence of four characteristic lines on the ESR spectrum. As shown in Fig. 2b, the characteristic ESR signals of BMPO/·OH were negligible in the absence of Tb_4_O_7_ NPs. However, the addition of Tb_4_O_7_ NPs resulted in a strong ESR spectrum that displayed the four characteristic lines (1:2:2:1) of BMPO/·OH. These results clearly show that Tb_4_O_7_ NPs can be used as a catalyst in the decomposition of H_2_O_2_ to produce hydroxyl radicals.

Taken together, our results confirm that Tb_4_O_7_ NPs are capable of catalyzing the oxidation of biologically relevant antioxidant agents, resulting in the production of H_2_O_2_. Moreover, Tb_4_O_7_ NPs can further catalyze the production of hydroxyl radicals via the decomposition of H_2_O_2_.

### Antibacterial activity of Tb_4_O_7_ NPs

**Fig. 3:**
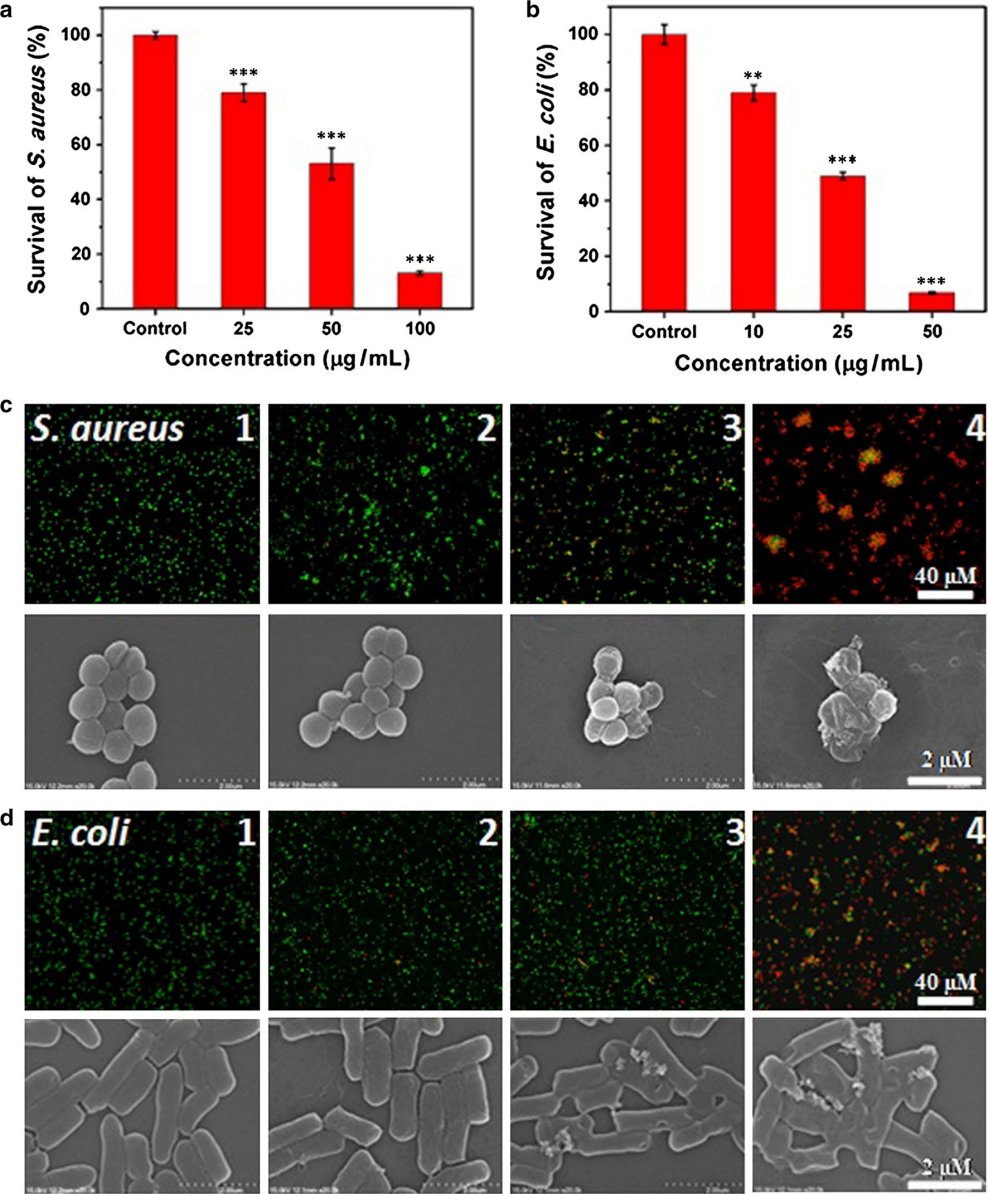
The effect of Tb_4_O_7_ NPs on survival rates of bacteria **a** CFUs of *S. aureus* following incubation with Tb_4_O_7_ NPs; **b** CFUs of *E. coli* following incubation with Tb_4_O_7_ NPs; **c** Representative fluorescence and SEM images of *S. aureus* after Tb_4_O_7_ NPs treatments. Bacterial cells were treated with 1) PBS as control, 2) 25 μg/mL Tb_4_O_7_ NPs, 3) 50 μg/mL Tb_4_O_7_ NPs or 4) 100 μg/mL Tb_4_O_7_ NPs; **d** Representative fluorescence and SEM images of *E. coli* after Tb_4_O_7_ NPs treatments. Bacterial cells were treated with 1) PBS as control, 2) 10 μg/mL Tb_4_O_7_ NPs, 3) 25 μg/mL Tb_4_O_7_ NPs or 4) 50 μg/mL Tb_4_O_7_ NPs. **p < 0.01 and ***p < 0.001 vs control

Both H_2_O_2_ and hydroxyl radicals have strong oxidizing ability and can oxidize biological macromolecules, such as proteins and phospholipids [32]. Our study found that Tb_4_O_7_ NPs with oxidase-like activity can catalyze the production of H_2_O_2_ and further produce hydroxyl radicals. Therefore, the oxidase-like activity of the Tb_4_O_7_ NPs makes them potentially useful as antibacterial agents. We evaluated the antibacterial activity of Tb_4_O_7_ NPs against *E. coli* and *S. aureus*. A colony-forming units plate counting method was used to determine the antibacterial ability (Fig. 3a, b). As compared to the PBS control group, Tb_4_O_7_ NPs exhibited potent antimicrobial activity against both *S. aureus* and *E. coli* in a concentration-dependent manner. At a concentration of 25 μg/mL, Tb_4_O_7_ NPs exhibited only modest antibacterial effects against *S. aureus*; more than 80% of the bacterial cells survived. However, when the concentration of Tb_4_O_7_ NPs was increased to 100 μg/mL, nearly 90% of the *S. aureus* were killed. A similar trend of antibacterial effects were observed towards the *E. coli*.

To further investigate the interaction between Tb_4_O_7_ NPs and bacteria, a fluorescent-based cell live/dead assay was conducted. As shown in Fig. 3c, d, Tb_4_O_7_ NPs exhibited significant antibacterial activity against both *S. aureus* and *E. coli*, which was consistent with the aforementioned results. The exposure of *S. aureus* cells to Tb_4_O_7_ NPs at a concentration of 100 μg/mL resulted in nearly 100% lethality, as evidenced by the dominant red fluorescent signal. At a concentration of 50 μg/mL, Tb_4_O_7_ NPs completely inhibited the bacterial growth of *E. coli*.

The morphology and membrane integrity of bacteria were then determined by SEM (Fig. 3c, d). Untreated *S. aureus* displayed a typical rod-shaped structure with a continuous, smooth surface. When exposure to the 50 μg/mL of Tb_4_O_7_ NPs, the *S. aureus* bacterial cell walls became partially wrinkled and discontinuance. Notably, after treatment with 100 μg/mL of Tb_4_O_7_ NPs, the *S. aureus* bacterial cell walls showed much more pronounced damage, indicating stronger antibacterial effects at higher concentrations of Tb_4_O_7_ NPs. A similar tendency was found for *E. coli*. The loss of membrane integrity of *E. coli* was observed at concentrations lower than 100 μg/mL of Tb_4_O_7_ NPs treatment. Moreover, Tb_4_O_7_ NPs were observed by SEM and SEM-energy dispersive X-ray spectroscopy (EDS) to aggregate on the surfaces of *S. aureus* and *E. coli* (Additional file 1: Figure S4).

**Fig. 4:**
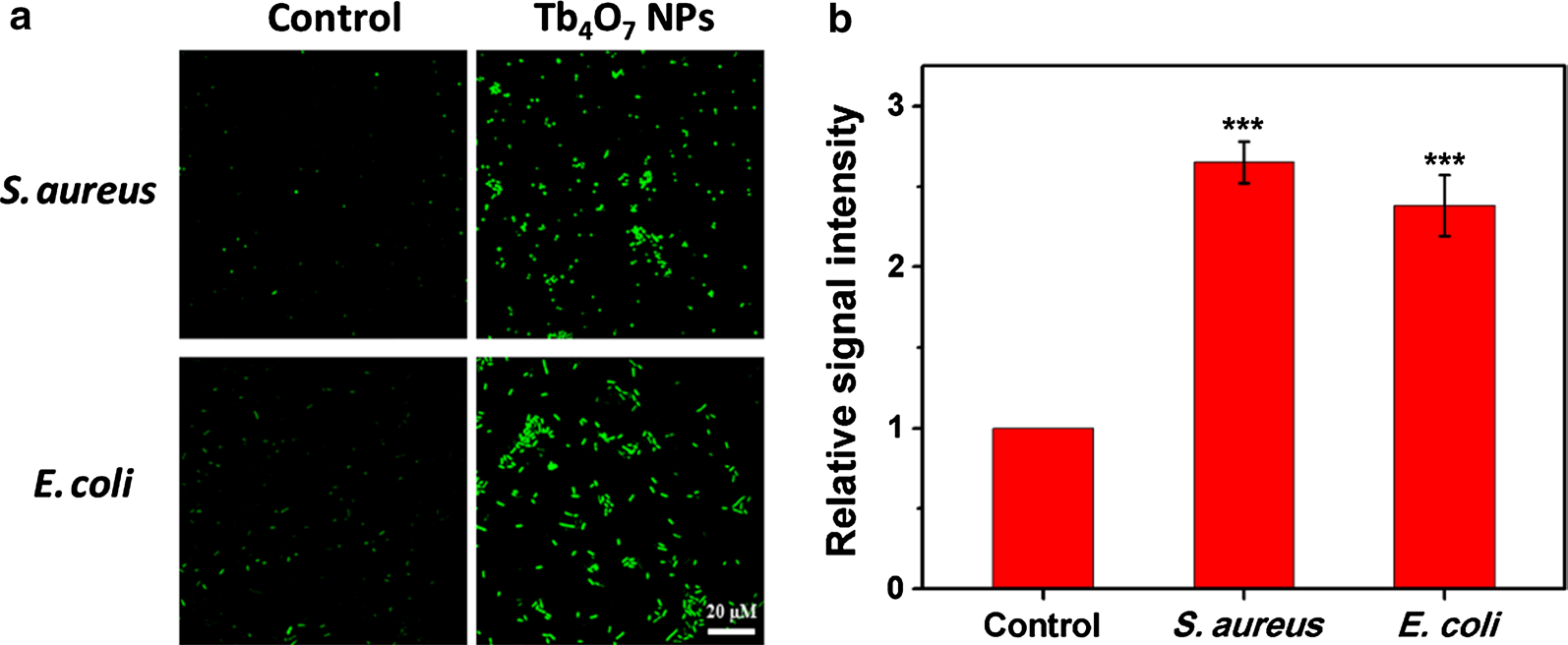
**a** Fluorescence images of bacterial cells. **b** Analysis of the ROS levels by microplate reader. ***p < 0.001 vs control

It is known that the proton motive force decreases the local pH (as low as pH 3.0) in the cytoplasm and membrane of bacteria cells [33, 34]. Since we found that Tb_4_O_7_ NPs exhibit oxidase-like activity under acidic conditions, we speculate that the antibacterial mechanism of Tb_4_O_7_ NPs may arise from their oxidase activity to accelerate the process of bacterial cell oxidation and consumption of antioxidant biomolecules, leading to a reduction of oxygen products including H_2_O_2_ along with other antibacterial activity from the accumulation of ROS. To confirm this hypothesis, the intracellular levels of ROS were determined using the florescent probe, DCFH-DA (Fig. 4). For both *S. aureus* and *E. coli*, the untreated cells showed extremely weak fluorescence, indicating low levels in the formation of intracellular ROS. In contrast, bacterial cells exposed to Tb_4_O_7_ NPs showed high levels of ROS formed within the cellular cytoplasm, as evidenced by the strong fluorescence signal. We also found that the generation of ROS is Tb_4_O_7_ NPs dose-dependent (Additional file 1: Figure S5).

### In vivo wound disinfection effect of Tb_4_O_7_ NPs

**Fig. 5:**
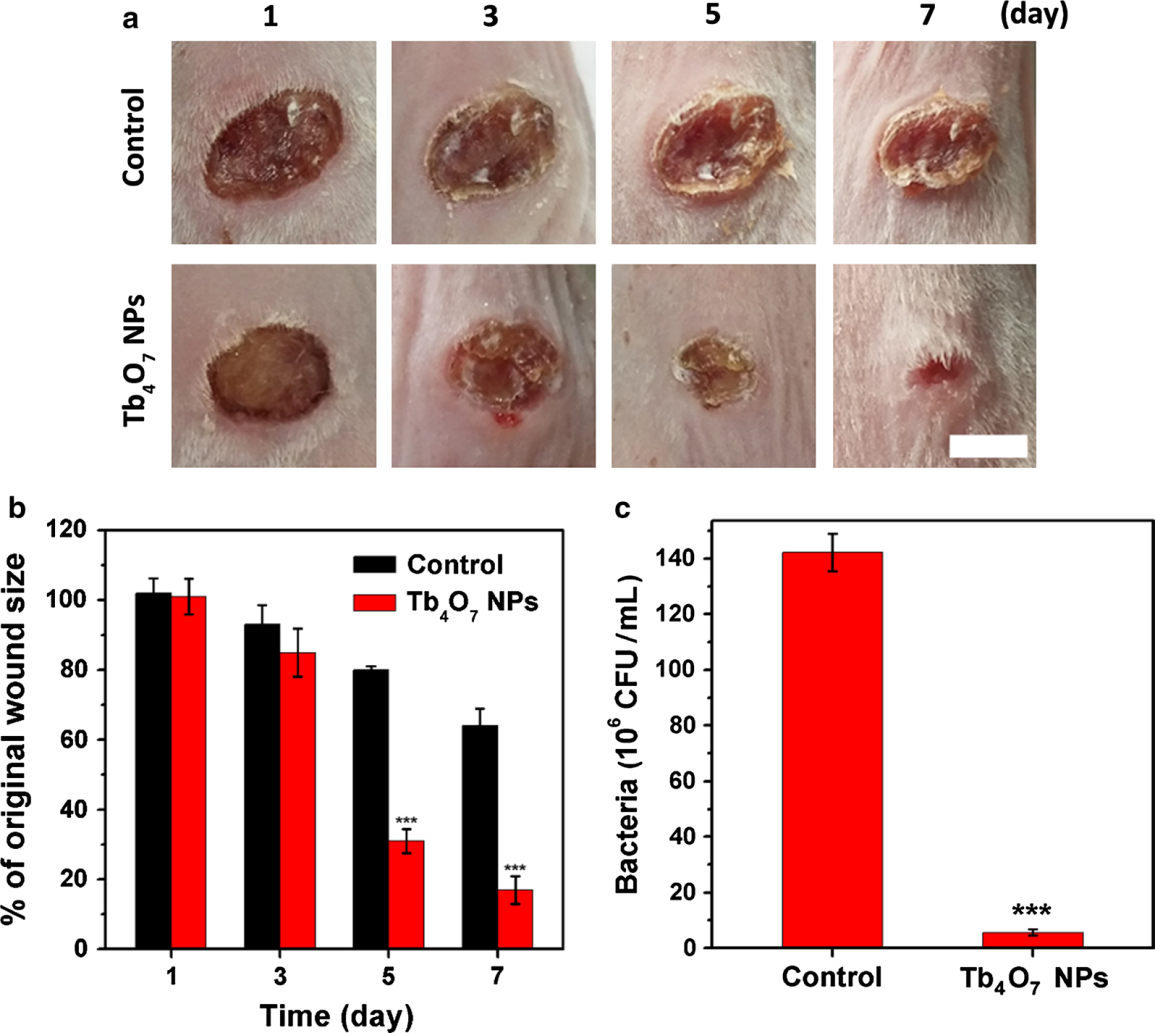
**a** Photographs of wounds on the backs of mice in control (PBS) and Tb_4_O_7_ NPs treatment groups (n = 5). Scale bar: 5 mm. **b** Related wound size in each treatment group. **c** Bacterial number of infected wounds on the 7th day. ***p < 0.001 vs control

The above findings suggest that Tb_4_O_7_ NPs may have role as an antibacterial agent for bacterial infections in vivo. To assess the antibacterial capacity of Tb_4_O_7_ NPs in vivo, a wound infection model was constructed using BALB/c mice. A wound was introduced on the back of the mouse and an infection was established by implanting *S. aureus* into the wounded area. After infection was established, PBS or Tb_4_O_7_ NPs were applied to the infected wound. Figure 5a, b shows the progress of the wounds. Compared with the control (PBS treatment) group after 3 days, the wound area was reduced under Tb_4_O_7_ NPs treatment. After treating of 7 days with Tb_4_O_7_ NPs treatment, the wounds were nearly healed completely. In contrast, obvious scab was observed from the control group, indicating incomplete recovery.

### Biosafety of Tb_4_O_7_ NPs

**Fig. 6:**
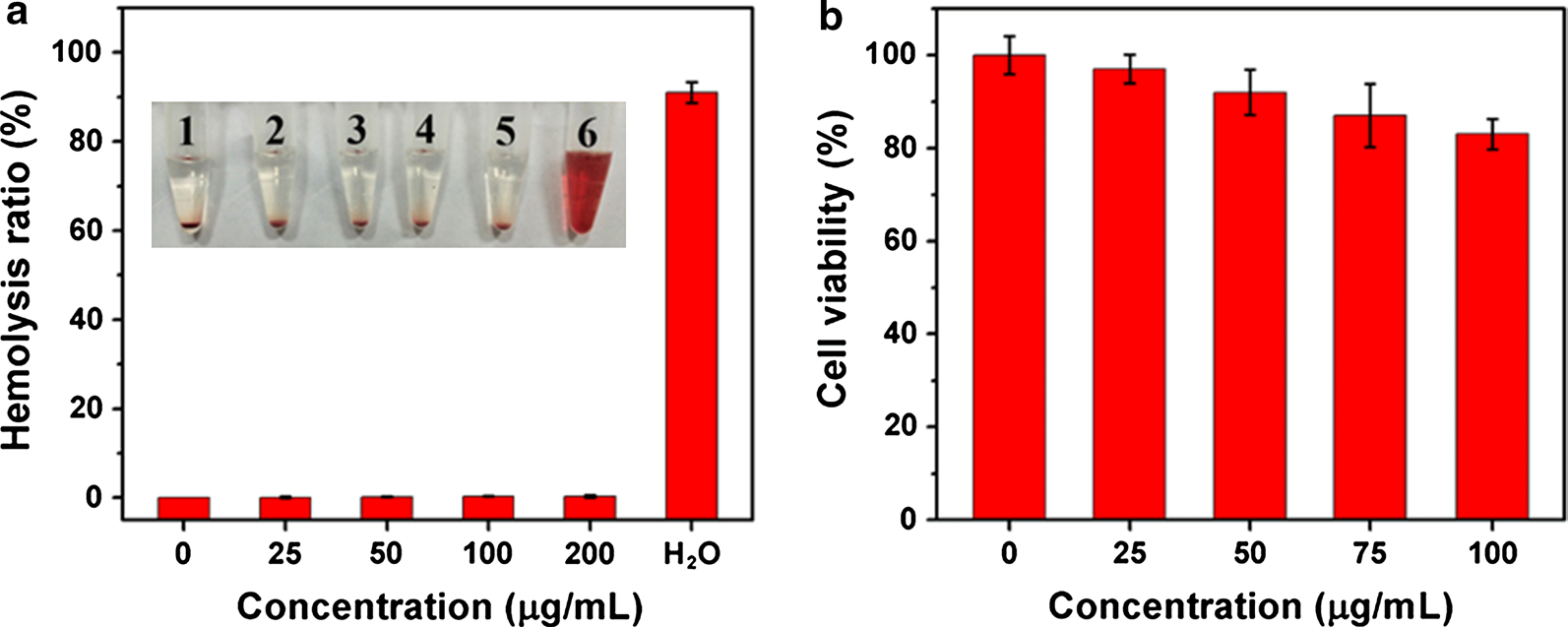
**a** The hemolysis ratio of red blood cells. The insert images of tubes containing red blood cells solution show the direct observation of hemolysis. Tube 1: PBS buffer; Tube 2–5: 25, 50, 100, and 200 μg/mL Tb_4_O_7_ NPs; Tube 6: ultrapure water. **b** MTT assays determined cell viability of HUVECs after Tb_4_O_7_ NPs treatment

Biosafety is an important factor for antimicrobial agents designers. To assess the biosafety of Tb_4_O_7_ NPs, we first determined the effects of Tb_4_O_7_ NPs on red blood cells and HUVECs in vitro. The effect of Tb_4_O_7_ NPs on cell membrane disrupt was first determined by a red blood cell hemolysis assay. As shown in Fig. 6a, pure water can cause severe red blood cells hemolysis within 2 h. In contrast, the introduction of Tb_4_O_7_ NPs did not cause signs of hemolysis. In this experimental result, the hemolysis rate was still less than 1% at a concentration of 100 µg/mL, which fully demonstrated that Tb_4_O_7_ NPs have good blood compatibility. Meanwhile, the cytotoxicity tests on mammalian cells HUVECs further confirm the biosafety of Tb_4_O_7_ NPs (Fig. 6b).

**Fig. 7:**
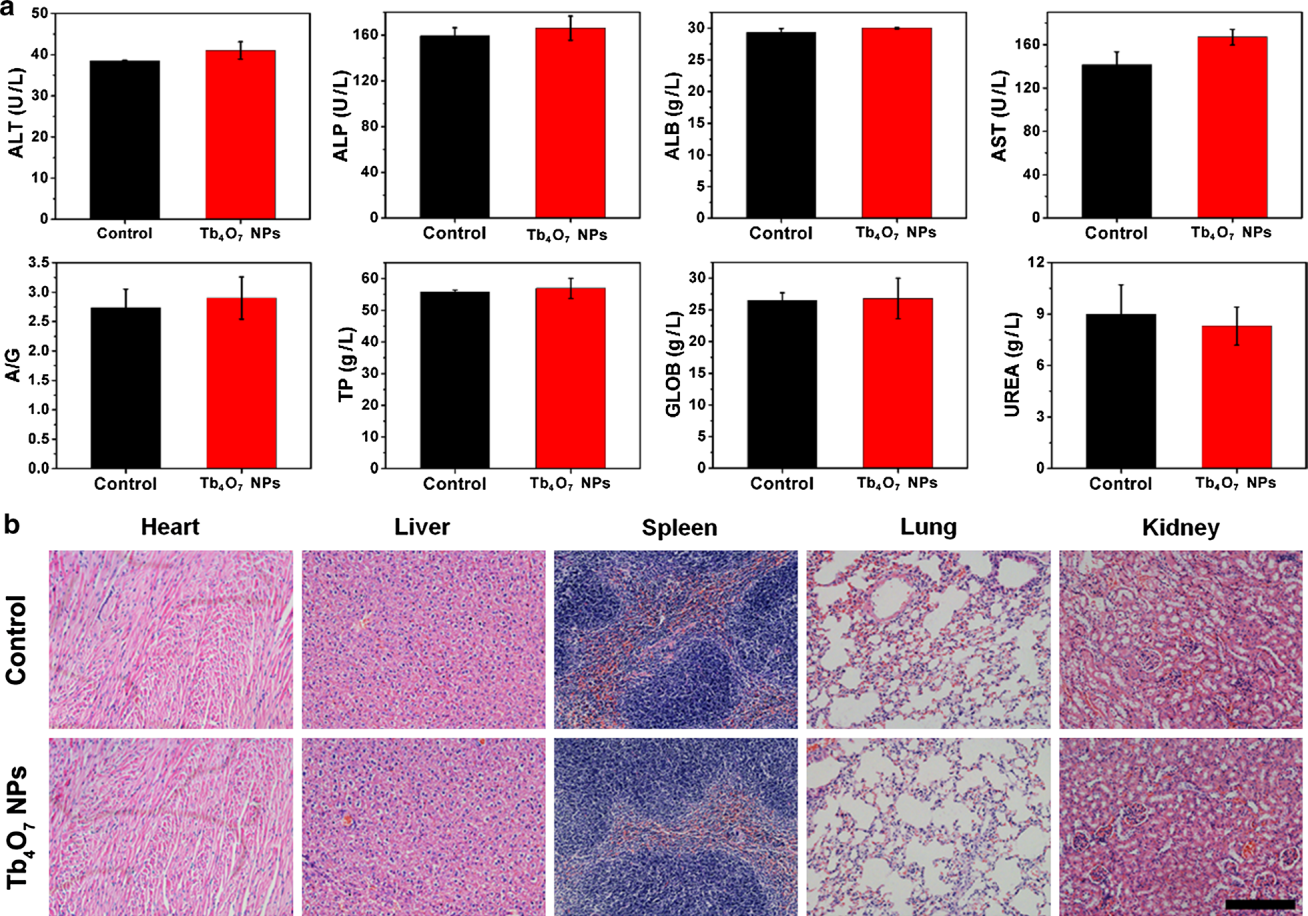
In vivo toxicity of Tb_4_O_7_ NPs. **a** The blood biochemistry data of the mice treated with Tb_4_O_7_ NPs after 7 d (n = 5). **b** Histological data (H&E staining images) are obtained from the major organs of mice treated with Tb_4_O_7_ NPs after 7 d. Scale bar = 100 μm

Then, the biosafety of Tb_4_O_7_ NPs in vivo were determined. As shown in Fig. 7a, the indicators in blood were within the normal range. The major mouse organs (heart, liver, spleen, lung, and kidney) were formalin-fixed and processed for the evaluation of H&E sections by histopathology (Fig. 7b). No obvious mouse organ damage was observed from Tb_4_O_7_ NPs treatment.

## Conclusions

In summary, this study demonstrates for the first time that Tb_4_O_7_ NPs exhibit oxidase-like activity. In addition, the results of this study established a relationship between the oxidase-like enzyme activity of Tb_4_O_7_ NPs and their antibacterial properties. The data collected from this work revealed that the oxidase-like activity of Tb_4_O_7_ NPs was able to function with a variety of substrates, including biomolecules, and resulted in the generation of ROS, which further enhanced their antibacterial activity. The application of the antibacterial activities of Tb_4_O_7_ NPs were demonstrated in a wound infection mouse model. Our study provides evidence that Tb_4_O_7_ NPs can be utilized as an efficient antibacterial agent and the potential applications in wound healing are promising.

## Additional file

**Additional file 1: Figure S1.** Characterization of Tb_4_O_7_ NPs. **Figure S2.** The oxidase-like catalytic activity of the Tb_4_O_7_ NPs. **Figure S3.** The concentration of H_2_O_2_ generated in the catalytic system. **Figure S4.** SEM-EDS elemental images. **Figure S5.** ROS levels of *S. aureus*.

## Abbreviations

Tb_4_O_7_: terbium oxide
NPs: nanoparticles
H_2_O_2_: hydrogen peroxide
ROS: reactive oxygen species
ABTS: diammonium 2,2′-azino-bis(3-ethylbenzothiazoline-6-sulfonate)
OPD: *o*-phenylenediamine
AA: ascorbic acid
DCFH-DA: 2′,7′-dichlorodihydrofluorescein diacetate
BMPO: 5-tert-butoxycarbonyl-5-methyl-1-pyrroline-*N*-oxide
CCK-8: Cell Counting Kit-8
*E. coli*: *Escherichia coli*
*S. aureus*: *Staphylococcus aureus* subsp. *aureus*
HUVEC: human umbilical vein endothelial cells
TEM: transmission electron microscopy
ESR: electron spin resonance
CFUs: colony forming units
PI: propidium iodide
SEM: scanning electron microscope
DLS: dynamic light scattering
EDS: energy dispersive X-ray spectroscopy
