# Supplementary material for: Bactericidal effects and accelerated wound healing using Tb4O7 nanoparticles with intrinsic oxidase-like activity
Source: J Nanobiotechnology. 2019 Apr 16;17:54. doi: 10.1186/s12951-019-0487-x (PMC6466657; doi:10.1186/s12951-019-0487-x)
Supplement: Supplementary file 1 — Additional file 1: Figure S1. Characterization of Tb4O7 NPs. Figure S2. The oxidase-like catalytic activity of the Tb4O7 NPs. Figure S3. The concentration of H2O2 generated in the catalytic system. Figure S4. SEM-EDS elemental images. Figure S5. ROS levels of S. aureus. [file 12951_2019_487_MOESM1_ESM.docx]

**Additional file**

**Bactericidal effects and accelerated wound healing using Tb_4_O_7_ nanoparticles with intrinsic oxidase-like activity**

Chen Li^1†^, Yurong Sun^1†^, Xiaoping Li^1†^, Sanhong Fan^1*^, Yimin Liu^2^, Xiumei Jiang^3^, Mary D. Boudreau^4^, Yue Pan^2*^, Xin Tian^5*^ and Jun-Jie Yin^3^

*^1^ School for Life Science, Shanxi University, Taiyuan 030006, China.*

*^2^ Guangdong Provincial Key Laboratory of Malignant Tumor Epigenetics and Gene Regulation, Department of Radiation Oncology, Medical Research Center, Sun Yat-Sen Memorial Hospital, Sun Yat-Sen University, Guangzhou, 510120, China.*

*^3^ Division of Analytical Chemistry, Office of Regulatory Science, Center for Food Safety and Applied Nutrition, U.S. Food and Drug Administration, College Park, Maryland 20740, United States.*

*^4^ Division of Biochemical Toxicology, National Center for Toxicological Research, U.S. Food and Drug Administration, Jefferson, Arkansas 72079, United States.*

*^5^ State Key Laboratory of Radiation Medicine and Protection, School for Radiological and Interdisciplinary Sciences (RAD-X), Collaborative Innovation Center of Radiation Medicine of Jiangsu Higher Education Institutions, Soochow University, Suzhou 215123, China.*

**Other Experiments**

**Oxidase-like activities measurements**

Kinetic measurements were carried out in time course mode by monitoring the absorbance change at 652 nm according to a previous report.^24^ Experiments were carried out by using Tb_4_O_7_ NPs in a reaction volume of 1 mL buffer solution (25 mM Na_2_HPO_4_, pH 2.0-10.0). The Michaelis–Menten constant was calculated using the Lineweaver–Burk plot: 1/_v_ = (*K*_m_/*V*_max_)/[S] + 1/*V*_max_, where *v* is the initial velocity, *V*_max_ is the maximal reaction velocity, and [S] is the concentration of substrate.

**Antibacterial activity of Tb_4_O_7_ NPs**

*E. coli* and *S.aureus* were grown in Luria-Bertani and [Trypticase Soy Broth](https://www.atcc.org/~/media/F11236DC0E36489ABFA10BF4A411C525.ashx) to reach the mid-exponential growth phase at 37 °C, respectively. Bacterial suspensions were centrifuged (5000 rpm, 5 min) and washed with PBS buffer. The suspensions were diluted to obtain cell counts of 10^9^ bacteria/mL, and the bacteria were incubated with different concentrations of Tb_4_O_7_ NPs (0-100 μg/mL) for 2 h. The survival rate of bacterial was determined by counting the number of colony forming units (CFUs). The antibacterial activity of Tb_4_O_7_ NPs was measured using a Live/Dead BacLight viability kit. After bacteria were treated with Tb_4_O_7_ NPs, the bacteria were stained with SYTO9 and propidium iodide (PI) for 30 min at dark. The bacteria cells were washed with PBS buffer, and samples were visualized using a confocal laser microscope (FV1200, Olympus, Japan).

**Cell morphology observation**

After antibacterial assessment, bacteria were harvested by centrifugation and fixed with 2.5% glutaraldehyde. The bacteria were gradually dehydrated in ethanol, and the dried bacteria were visualized using a scanning electron microscope (SEM, S-4700, Hitachi, Japan).

**In vivo toxicity study**

A total dose of 20 μg Tb_4_O_7_ NPs (100 μg/mL in PBS buffer) were subcutaneously injected into BALB/c mice (n = 5). After the 7th day of treatment, the major organs were collected under sterile conditions from mice for pathological examination.

**
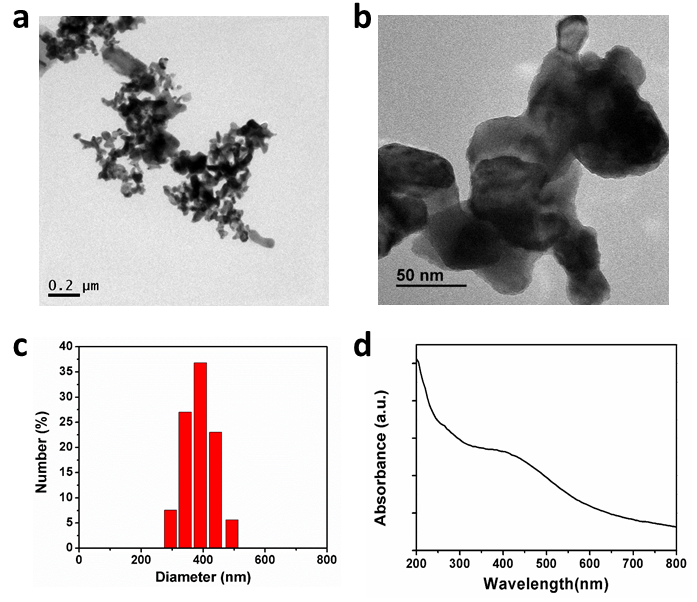
**

**Fig. S1** Characterization of Tb_4_O_7_ NPs. (**a,b**) TEM images of Tb_4_O_7_ NPs. (c) DLS data of Tb_4_O_7_ NPs. (**d**) UV-vis spectrum of Tb_4_O_7_ NPs.


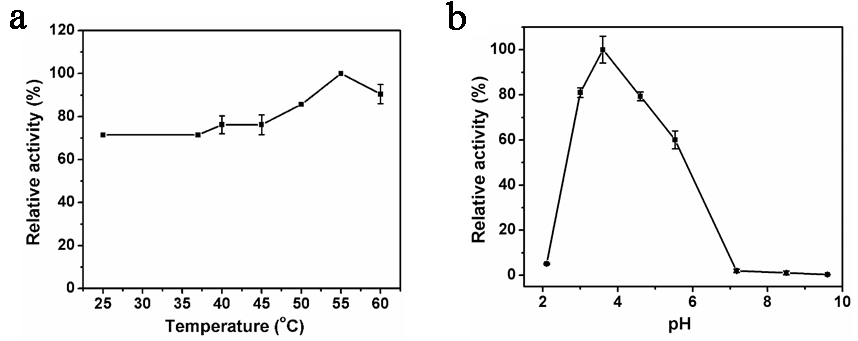


**Fig. S2** The oxidase-like catalytic activity of the Tb_4_O_7_ NPs against temperature (**a**) and pH (**b**). Error bars indicate the standard deviations of three independent measurements.





**Fig. S3** The concentration of H_2_O_2_ generated in the catalytic system: AA alone (control) and AA with the different concentrations of Tb_4_O_7_ NPs. Error bars indicate the standard deviations of three independent measurements. **p* < 0.05 and ***p* < 0.01, indicting significantly the statistical difference compared with control.


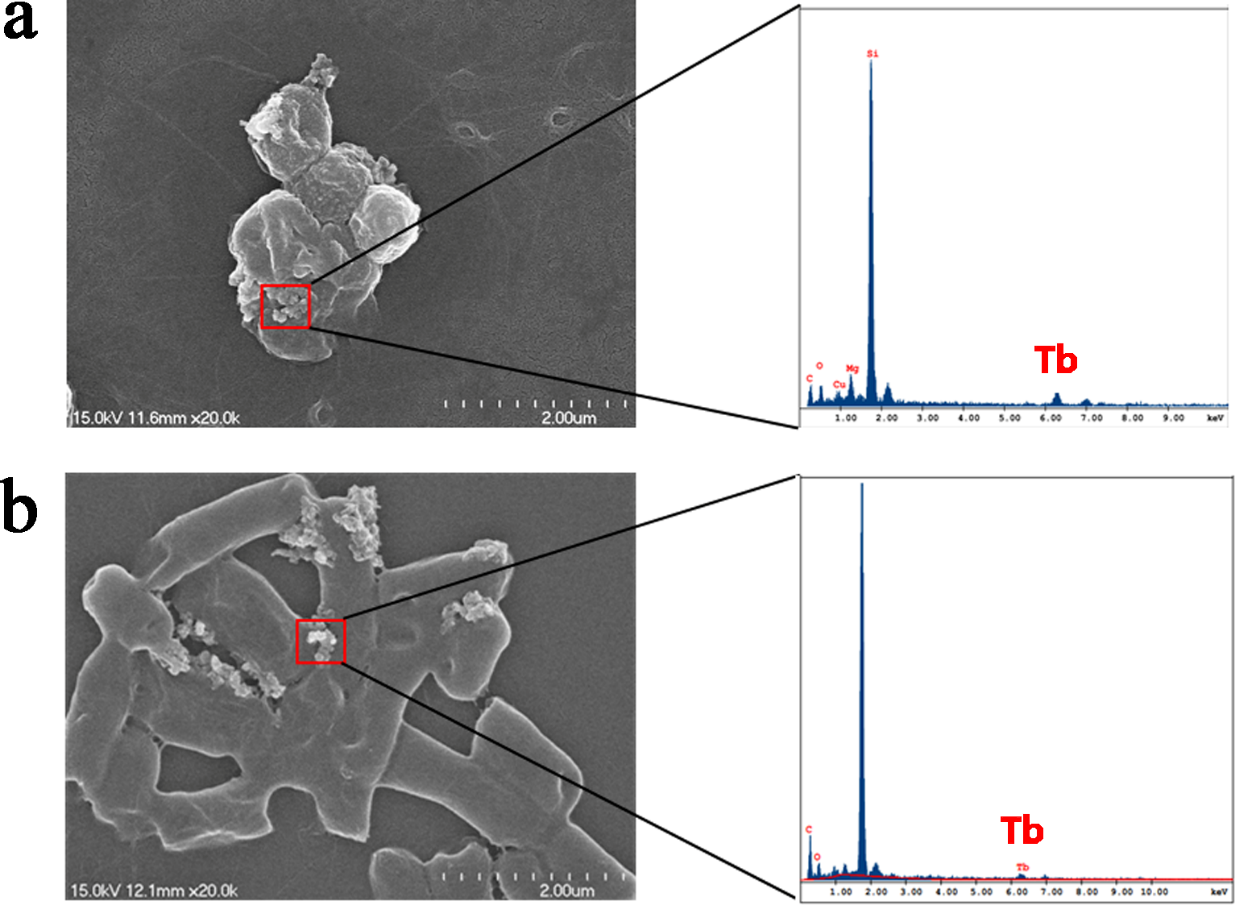


**Fig. S4** SEM-EDS elemental images of *S. aureus* (**a**) and *E. coli* (**b**) cells exposed to Tb_4_O_7_ NPs.

.



**Fig. S5** Analysis of the ROS levels of *S. aureus* after treatment by different concentrations of Tb_4_O_7_ NPs. ***p* < 0.05 and ****p* < 0.001 vs control.
